# Therapeutic exercise to improve motor function among children with Down Syndrome aged 0 to 3 years: a systematic literature review and meta‑analysis

**DOI:** 10.1038/s41598-022-16332-x

**Published:** 2022-07-29

**Authors:** Eliana-Isabel Rodríguez-Grande, Adriana Buitrago-López, Martha-Rocio Torres-Narváez, Yannely Serrano-Villar, Francisca Verdugo-Paiva, Camila Ávila

**Affiliations:** 1 Master′s and PhD programs in Clinical Epidemiology, Pontificia Universidad Javeriana, Bogotá, Colombia; 2 School of Medicine and Health Sciences, Universidad del Rosario, GI Rehabilitation Sciences, Carrera 24 N. 63D – 69, PBX 2970200 Ext. 3420, Bogotá, Cundinamarca Colombia; 3 Erasmus University Rotterdam, Rotterdam, The Netherlands; 4 Universidad Católica de Paraná, Curitiba, Brazil; 5 Department of Clinical Epidemiology and Biostatistics, School of Medicine, Pontificia Universidad Javeriana, Bogotá, Colombia; 6 School of Medicine and Health Sciences, Universidad del Rosario, GI Rehabilitation Sciences, Physiotherapy Program, Bogotá, Colombia; 7 Universidad CES, Medellín, Colombia; 8 Centro Evidencia UC, School of Medicine, Pontificia Universidad Católica, Santiago de Chile, Chile; 9 Fundación Epistemonikos, Santiago de Chile, Chile

**Keywords:** Health care, Health occupations, Medical research

## Abstract

The effects and the prescription parameters of therapeutic exercise are not clear. For this reason, is needed to determine the effect of therapeutic exercises on the motor function of children with Down Syndrome (DS) aged 0 to 3 years. The present study is systematic review and meta-analysis of effectiveness outcomes in this population: gait, balance, motor development, fine motor skills, and executive functions. The databases of PubMed, PEDro, EMBASE, SCIELO, Lilacs, Cochrane library were searched from January to December 2019. We recruited Randomized Controlled Trials (RCTs) which met the inclusion criteria in our study. Six studies and 151 participants were included. Two types of therapeutic exercises, aerobic and neuromuscular, were identified. Both types of exercise were effective in improving outcomes. There were no differences between the modes of application of the exercise. No differences were identified between the treadmill and the physiotherapy plan for the reduction of the time to reach independent walking, Mean Difference (MD) 46.79, 95% Confidence Interval (IC) (− 32.60, 126.19), nor for the increase in walking speed MD 0.10 IC (− 0.02, 0.21) m/s. This study suggests that aerobic exercise therapy has a potentially effective role to promote the gait and motor development of children with DS aged 0 to 3 years when it is applied using a treadmill with a frequency of 5 days, a duration of 6–8 min, and an intensity of between 0.2 and 0.5 m/s. Studies with less heterogeneity and larger sample sizes are required.

## Introduction

Down Syndrome (DS) in children triggers musculoskeletal and intellectual disorders, which in turn impact motor function^1^. This function encompasses the set of motor skills whose execution can be observed and measured in terms of orientation, displacement, speed, and acceleration and also includes coordinated and intentional actions that are part of the child’s daily interactions, such as crossing obstacles and walking over irregular terrains^2^. The motor function also includes those internal processes associated with practice, experience, and the context in which movement develops, which enable the learning process needed to acquire a specific motor skill^3–5^.

The acquisition of gross and fine motor functions in children with DS is different compared with that in typically developing children, mainly due to the level of development of the central nervous system, hypotonia, the presence of primitive reflexes, and joint hypermobility ^(4,6,7).^ Children with DS generally start walking around age 3 and have a limited performance regarding balance, hand–eye and foot–eye coordination, and the speed at which they can execute fast movement patterns, such as jumping or kicking1). This leads to difficulties in developing activities of daily living^6,8^ and poses a challenge for their caregivers, considering that only 11.6% of 5-year-old children with DS can brush their teeth and 0% can tie their shoes^8,9^.

Children with DS require health care plans that promote a better motor skill performance and that include therapeutic interventions, such as therapeutic exercises^10^. Therapeutic exercise is a physical activity that can be measured accurately and responds to therapeutic objectives according to the patient’s condition^11,12^. Physical exercise applied to the clinical setting contributes to promoting, enhancing, or restoring physical health and musculoskeletal function and may have a positive impact on any of the body systems^12,13^.

Prescription parameters of therapeutic exercise must include the type, mode, frequency, intensity, and duration of the exercise^12^. The recommended dose of exercise under the prescription parameters should be sufficient to achieve the proposed therapeutic goals^12,14^. Currently, the combination of these prescription parameters has generated multiple doses of exercise, whose effectiveness in this population is not yet known^12,14^.

Literature has not reported a standard intervention yet or the application parameters that have been proven to be effective in enhancing these children’s motor function. Considering the complexity of the motor function construct, which includes a significant number of outcomes, there is lack of accurate knowledge on which of them should be promoted or on the most appropriate therapeutic interventions to do so.

No systematic reviews assessing the effects of therapeutic exercise on children with DS during the first years of life have been identified in the reviewed literature. A significant number of systematic reviews on exercise in individuals with DS have been published. These reviews focused on the juvenile or adult stage of this population^15,16^, mainly assessing cardiovascular outcomes^17,18^. In childhood researches, there are also some systematic reviews on the effects of exercise. However, these publications have studied the effects of therapeutic aerobic exercise on children with intellectual disabilities^19^, including those with other conditions that are accompanied by an intellectual disability, such as cerebral palsy or autism^20,21^.

There are multiple interventions that can be carried out to improve the motor function of children with DS, which hinders clinical decision-making when the parameters that have been proven to be effective in the target population of this review are not clear^22,23^. Likewise, the effective parameters of different types of exercises other than aerobic exercises in children with DS also remain unclear. Therefore, it is necessary to determine the effect of therapeutic exercise on the motor function of children with DS aged 0 to 3.

## Materials and methods

This review was performed in accordance with relevant guidelines and regulations. The preferred reporting items for systematic reviews and meta-analysis (PRISMA) guidelines were followed to assure transparent reporting^24^. This analysis was prospectively registered on Open Science Framework (OSF) and it is available in https://doi.org/10.17605/OSF.IO/WZQXT. Ethical and internal review board approval was not required for this analysis as no human or animal subjects were involved.

### Eligibility criteria

Participants: children with DS aged 0–3 years, bearing in mind that, during this period, the interventions reported in the literature aim to enhance the occurrence of motor patterns in children with DS. After age 4, the therapeutic goal is mainly focused on enhancing or rehabilitating the motor functions^10^.

#### Interventions

The study included all the therapeutic interventions that are duly applied and systematically planned physical exercises with specific prescription parameters in terms of intensity, frequency, and duration, among others, with the aim of promoting, improving, or maintaining the motor function of children with DS. Subsequently, long-duration interventions, in which the work of large muscle groups were promoted, were classified as aerobic exercise^25^. Therapeutic neuromuscular exercise was classified as the exercise aimed at improving the participants’ balance or flexibility and as resistance training, short-duration exercises whose energy system was mainly anaerobic.

Comparison between aerobic exercise (treadmill) and activities of daily living or physiotherapy plan also were compared exercise prescription parameters.

#### Outcomes

The reviewed literature showed publications on the key outcomes of —gait, balance, motor development, fine motor skills, and executive functions^26^.

*Study design* A literature systematic review was carried out, including Randomized Controlled Trials (RCTs).

#### Exclusion criteria

*Texts not available in full text* study authors were contacted to provide full text. If no response was obtained, the study was excluded.

### Search and identification of studies

The search strategy was designed based on the Population, Intervention, Comparison and Outcome (PICO) elements of the questions asked. These terms were adapted according to the languages of the different databases explored. A systematic search was conducted from January to December 2019, on databases such as PubMed, PEDro, EMBASE, SciELO, Lilacs, and the Cochrane Library. Additionally, other sources of evidence were consulted to allow the identification and analysis of published and unpublished literature (gray literature) that would not have been detected through a systematic search. Manual searches were conducted in the documents found in the reference lists and in journals specialized in the subject. In addition, Epistemonikos was consulted for previous systematic reviews on this topic in order to review the primary studies included in them, and an evidence-based matrix was built based on this information. This process was developed during the months of January to December 2019.

*The terms used included* Down syndrome, mongolism, trisomy, child, therapeutic exercise, exercise, aerobic, resistance training, physical therapy, physical, activity, therapeutic, resistance training, plyometric, stretching, anaerobic, bicycling, aquatic, rehabilitation, kinesiotherapy.

### Selection of studies

Study selection based on titles and abstracts was performed independently by two trained reviewers (EIRG and YSV). RCTs that assessed the effectiveness of therapeutic exercise and reported the effectiveness in the outcomes—selected.

Each assessor generated BibTeX files of the selected studies. Using a bibliographic manager, duplicates were regarded as studies with agreement between the assessors, and those that were not duplicated were reviewed individually by the two assessors and their eligibility was discussed and determined. The eligibility of those studies without a discussion-based consensus was decided by a third assessor.

Studies that did not include at least one of the outcomes or applied a combination of therapeutic exercise interventions and pharmacological interventions were excluded from the study.

### Data collection process

Data were extracted through pre-designed data collection formats. The data from the variables were collected for the comparison of the studies and the measurement of outcomes.

For the gait outcome, the data such as time-to-event or changes in the kinematic or kinetic parameters of this variable were extracted. For the balance outcome, the data on displacement of the center of mass or time maintaining postural balance were extracted. The independent variable comprised the type, mode, frequency, intensity, duration of the interventions, place of performance of the interventions (i.e., outpatient consultation or home) and the person in charge of applying the intervention (i.e., physiotherapist, other professional, family member, or caregiver).

Further data extracted from the population were age, sex, sample size for each group, and cognitive impairment.

### Assessment of study quality

Two independent assessors evaluated the risk of bias for each study using the Cochrane Collaboration tool^28^. The risk was assessed as *low risk of bias*, *high risk of bias*, and *unclear risk of bias* taking into account six domains: random sequence generation (selection bias), allocation concealment (selection bias), participant and staff blinding (performance bias), blinding of outcome assessment (detection bias), incomplete outcome data, and selective outcome reporting (reporting bias). The rating of risk of bias was assessed using the RevMan 5.1 software^29^.

### Synthesis of data

The selected body of evidence was assessed by prioritized outcomes. Each outcome described the population’s features; the parameters of the interventions including the exercise mode applied, frequency, intensity, and duration of the interventions applied in the said studies; and the quantitative results achieved with their level of significance, shown in Table 1. The data were synthesized on a Microsoft Excel base, extracting data from the population’s features, randomization methods, outcome measures, duration of follow-up, and assessment methods from each study. The meta-analysis considered direct comparisons between the experimental group who did the interventions (aerobic exercise and resistance exercise) and a control group who performed educational activities, recreational activities, or continuity with activities of daily living or interventions other than those of interest for this review.

**Table 1:** Primary studies and reports.

References of the studies included in this review:	Reports linked to the studies included in this review:
1. Looper, Ulrich (2010) Effect of treadmill training and supramalleolar orthosis use on motor skill development in infants with Down syndrome: a randomized clinical trial^33^	Looper, Ulrich (2011) Does orthotic use affect upper extremity support during upright play in infants with down syndrome?^34^
2. Wu, Looper, Ulrich, Ulrich, Angulo-Barroso (2007). Exploring effects of different treadmill interventions on walking onset and gait patterns in infants with Down syndrome^35^	Wu, Looper, Ulrich, Angulo-Barroso (2010). Effects of various treadmill interventions on the development of joint kinematics in infants with Down syndrome^36^ Angulo-Barroso, Wu, Ulrich. (2008). Long-term effect of different treadmill interventions on gait development in new walkers with Down syndrome^37^ Ulrich, Lloyd, Tiernan, Looper, Angulo-Barroso (2008). Effects of intensity of treadmill training on developmental outcomes and stepping in infants with Down syndrome: a randomized trial^38^
3. Angulo-Barroso, Burghardt, Lloyd, Ulrich (2008). Physical activity in infants with Down syndrome receiving a treadmill intervention^19^	Lloyd, Burghardt, Ulrich, Angulo-Barroso (2010). Physical activity and walking onset in infants with Down syndrome^39^
4. Harris (1981). Effects of neurodevelopmental therapy on motor performance of infants with Down’s syndrome^40^	
5. Ulrich, Ulrich, Angulo-Kinzler, Yun (2001). Treadmill training of infants with Down syndrome: evidence-based developmental outcomes^41^	
6. Lowe, McMillan, Yates (2015). Body Weight Support Treadmill Training for Children With Developmental Delay Who Are Ambulatory^42^	

Averages and standard deviations of the data available from the selected studies were extracted from the prioritized outcomes included in the studies. When the studies reported standard errors of the mean, the standard deviations were obtained by multiplying standard errors of the mean by the square root of the sample size. Standardized Mean Differences (SMDs) and 95% Confidence Intervals (95% CI) were calculated to combine the results of the studies using different measures for the same concept or of studies presenting variability in its features.

Heterogeneity between trials was assessed using the chi-squared test, a significance value of *p* < 0.05 after due consideration of the value of I^230^. Heterogeneity was reported as low (I^2^ = 0–25%), moderate (I^2^ = 26–50%), or high (I^2^ > 50%)^30^. The results were combined using the random effects model and the 95% CI was calculated. All of the above were carried out with the RevMan 5 software^29^.

### Assessment of the certainty of evidence

The assessment of the certainty of the evidence found was carried out using the GRADE approach^27^. The evidence found for each of the outcomes was rated considering the risk of bias, inconsistency, direct or indirect evidence and imprecision, the risk of selective outcome reporting, and the dose–response gradient. These outcomes were classified using a three-level ordinal scale that included *very serious, serious,* and *not serious,* except for the risk criteria for selective outcome reporting (*not detected* or *strong suspicion*), the size of the effect (*no effect, large,* or *very large)*, the confounding factors (*no effect, it would reduce the effect demonstrated,* or *suggest spurious effect*), and the dose–response gradient (*no* or *yes*), in which nominal and ordinal scales with other levels were used^31^.

## Results

### Selection of studies

**Figure 1:**
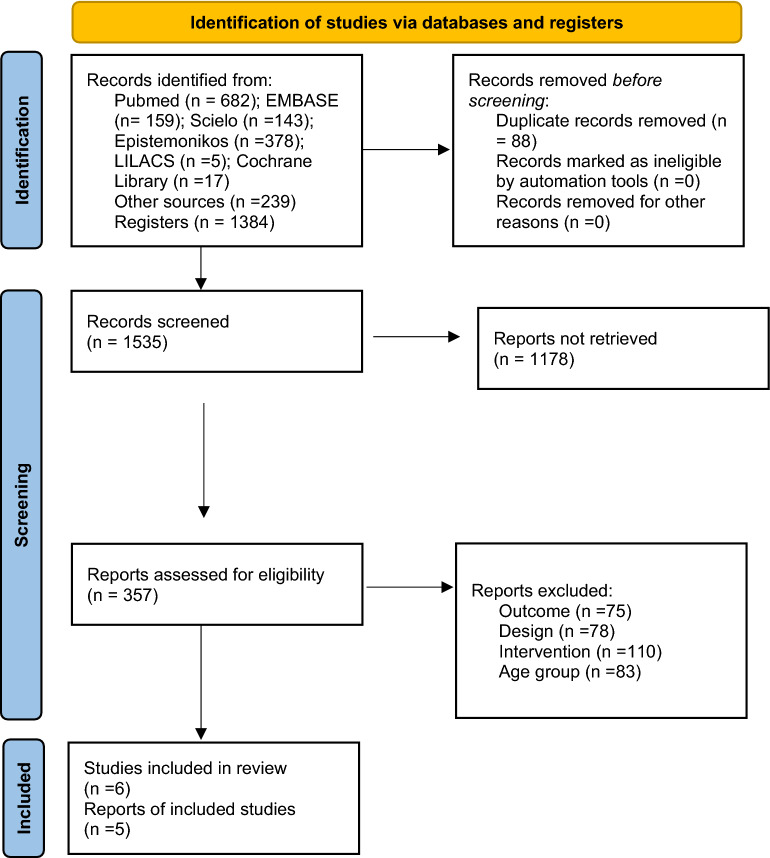
PRISMA flowchart of selection of articles included in the systematic review. Adapted from: Page MJ, McKenzie JE, Bossuyt PM, Boutron I, Hoffmann TC, Mulrow CD, et al. The PRISMA 2020 statement: an updated guideline for reporting systematic reviews. BMJ 2021;372:n71. https://doi.org/10.1136/bmj.n71. For more information, visit: www.prisma-statement.org.

A total of 1384 studies were found as a result of the systematic literature search. 239 studies were found in other sources that included the bibliographic references of the studies found in the systematic search and in those provided by the group of experts, amounting to a total of 1623 identified studies. Of these studies, 88 duplicated ones were excluded and 1178 studies were excluded considering the review of the titles and abstracts. The two assessors reviewed a total of 357 full-text studies, of which 347 were excluded because they did not meet the eligibility criteria, mainly due to the type of design, and because they did not include any of the prioritized outcomes for the systematic review. The flow chart of the studies found and included in the body of evidence is presented in Fig. 1.

Finally, six primary studies reported in eleven journals (*thread*) were included. Table 1 shows thread articles, primary studies, and reports linked to them^32^.

### Assessment of the risk of bias of the studies included

**Figure 2:**
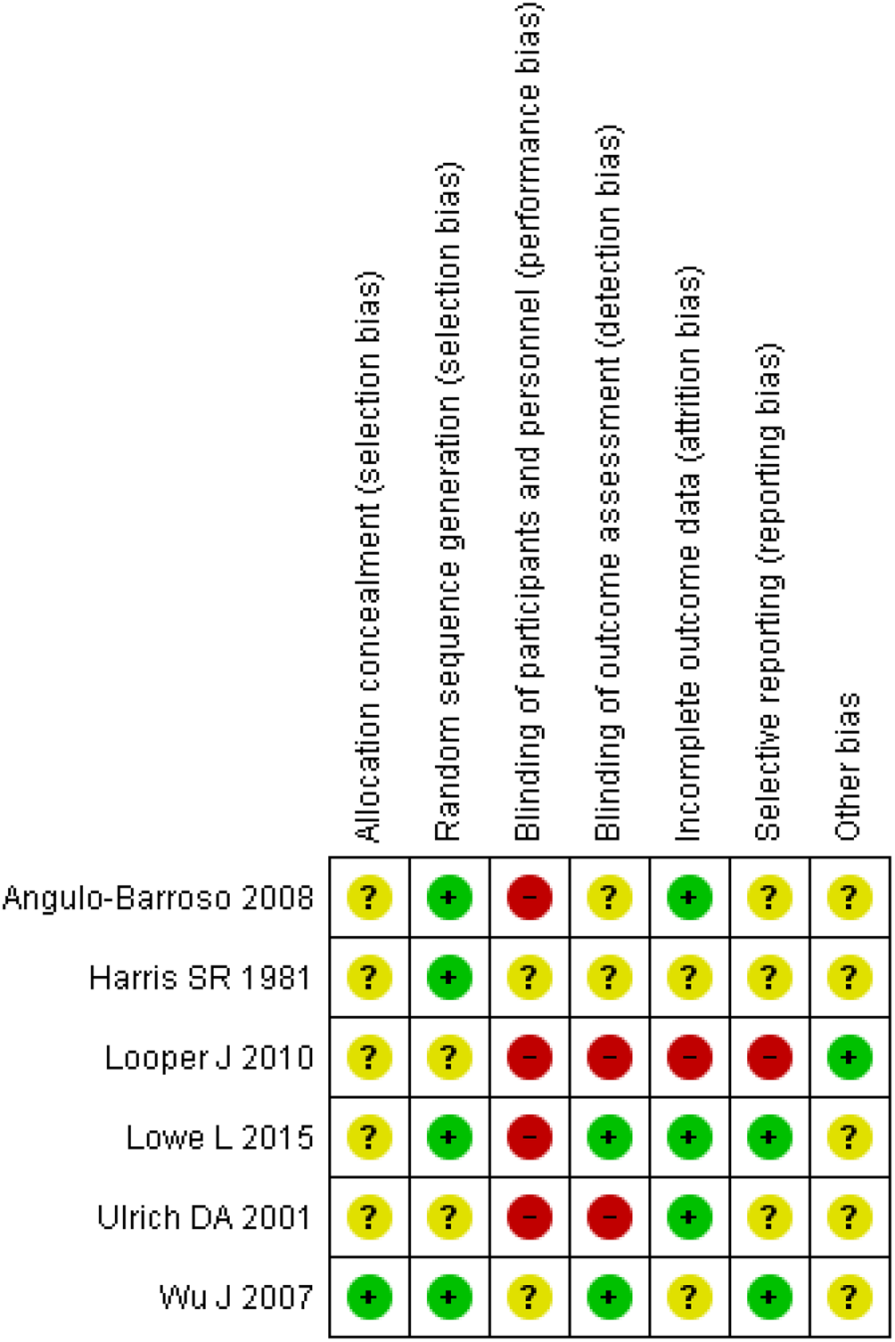
Risk of bias summary: review authors’ judgments on each risk of bias item for each included study. Revman 5. https://training.cochrane.org/online-learning/core-software-cochrane-reviews/revman.

**Figure 3:**
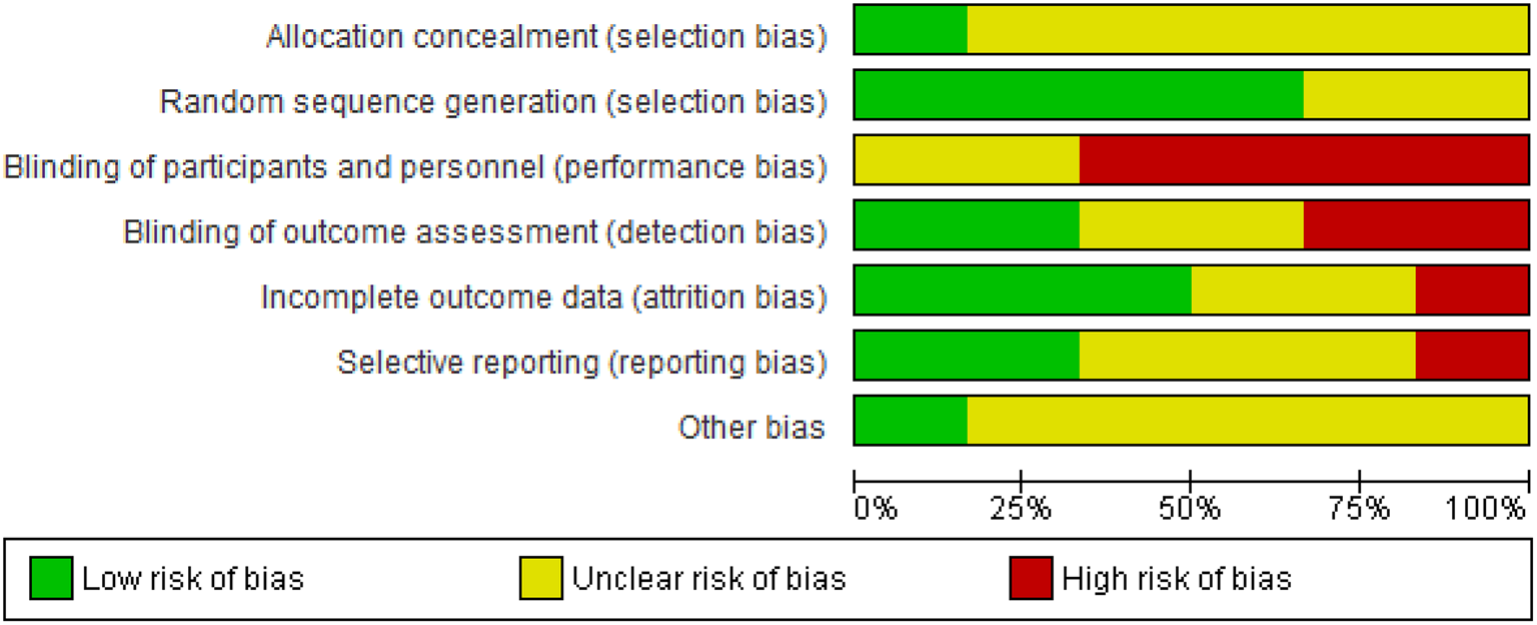
Risk of bias graph: review authors' judgments on each risk of bias item presented as percentages across all included studies. Revman 5. https://training.cochrane.org/online-learning/core-software-cochrane-reviews/revman.

Studies have less risk of bias in random sequence generation (70%) and more risk of bias in blinding of participants and personnel (70%) (Figs. 2 and 3).

#### Blinding

Due to the nature of the interventions used, the assessment of the risk of bias took into account the masking of outcomes by the assessors in each study^19,33,35,40,41^.

#### Selective reporting

One study was found to present high risk of bias^33^. The time of the independent walking event is considered an important outcome; nonetheless, the authors did not clearly report the time elapsed from the commencement of the study to the event of interest. Additionally, some data such as the analysis of video recordings collected during follow-ups were not reported.

#### Other potential sources of bias

None of the studies included in the review clearly mentioned the training processes of the outcome assessors or the adjustment and calibration processes of the equipment used, which is the reason why all of the studies, with the exception of the Looper study, were considered to have unclear risks of bias^33^.

### Types of therapeutic exercise and modes of application in physiotherapy interventions in children aged 0 to 3 years

In the literature included, only two types of therapeutic exercises were reported; the first one was classified as aerobic exercise as it included longer-duration interventions that promoted the work of large muscle groups^25^. Five of six studies included in this review applied this type of exercise and all coincided in the way the exercise was applied, by using the treadmill^19,33,35,41,42^.

The second type of exercise identified was neuromuscular, namely, the exercise that aims to improve the balance or flexibility of the participants. It mainly includes unstable surface activities^25^. The study by Harris SR was the only one including this type of exercise^40^. Table 2 includes the characteristics of the interventions along with their respective application parameters.

**Table 2:** Characteristics of the studies included regarding therapeutic exercise in children with DS aged 0 to 3 years.

Reference	Participants	Interventions	Outcomes, measurement tools	Application parameters	Results
Looper, J 2010^36^	DI: NR N: 17 H:NR M:NR Age 21.4 ± 4 months	EG: Treadmill and orthosis (eight hours a week), regular physiotherapy CG: treadmill, regular physiotherapy	* Independent gait: days to the event (gait: 3 independent steps) * Motor function: GMFM	**Type:** aerobic **Mode:** Treadmill **Frequency**: 5 days a week **Duration:** 8 min a day **Intensity:** Treadmill speed 0.2 m/s **Intervention applied by:** parents at home	**Average time to event: independent gait (three consecutive steps without assistance)** EG: 206 ± 109 days from the beginning of the intervention until the event CG: days to the event 268 ± 88 **Motor function** Higher scores in the group without orthosis at one-month follow-up *p* < 0.01 EG: GMFM pos 195.65 ± 8.12 CG: GMFM pos 183.78 ± 7.22
Wu, J 2010^35^	DI: NR N:30 H:18 M:12 Age 10.4 ± 2.14 months	EG: Treadmill at generally low intensity **Frequency**: 5 days a week **Duration:** 6 min a day **Intensity:** Treadmill speed 0.18 m/s CG: Treadmill at individualized high intensity **Frequency**: 5 days a week **Duration:** 8 min a day **Intensity:** Treadmill speed 0.5 m/s	**Independent gait**: Step length, stride length, speed **Motor development:** Bayley Scales for Infant Development	**Type:** aerobic **Mode:** Treadmill **Progression:** high intensity group: Treadmill duration and speed with ankle weights **Intervention applied by:** parents at home	Differences favoring group 1, both in months to the event and in gait parameters p < 0.05 EG: 19.2 months from the beginning of the intervention until event CG: 21.4 months to event
Angulo-Barroso, R. 2008^43^	DI: NR N:30 H:18 M:12 Age 10.4 ± 2.2 months	EG: Treadmill at generally low intensity CG: Treadmill at individualized high intensity	* Independent gait: days to the event (gait: 3 independent steps) *Parameters	**Type:** aerobic **Mode:** treadmill **Frequency:** 5 days a week **Duration:** 6 min a day **Intensity:** speed 0.18 m/s-0.22 m/s **Intervention applied by:** health professional	EG significant differences in time to event and in the development of the kinematic parameters of gait compared to CG p < 0.05
Harris, S. R.1981^40^	DI: NR N:20 H:9 M:11 Age 10.91 ± 7.64 months	EG: neurodevelopmental therapy CG: activities of daily living	* Motor development Bayley and Peabody Scales (fine and gross motor skills)	**Type:** neuromuscular **Mode:** Specific neurodevelopmental techniques that included joint approaches and resisted movements for postural tone, protective reactions and balance in supine and quadruped, rolling and creeping **Frequency**: 3 times a week for 9 weeks **Duration:** 40 min a day **Intensity:** NR **Intervention applied by**: parents at home	There were no significant differences between the groups, however, the experimental group showed significant differences between the initial and final measurement
Ulrich, D. A 2001^41^	DI: NR N:30 H:NR M:NR Age 9.2 ± 0.5 months	EG: Treadmill and comprehensive physiotherapy GC: comprehensive physiotherapy	**Independent gait**: time to event **Motor development:** Bayley Scales for Infant Development	**Type:** aerobic **Mode:** Treadmill **Frequency**: 5 days a week **Duration:** 8 min a day **Intensity:** Treadmill speed 0.2 m/s **Intervention applied by:** parents at home **Traditional physiotherapy:** health professional	**Independent gait** EG: 73.8 days from the beginning of the intervention until the event CG: days to event 101 days
Lowe L 2015^42^	DI: NR N: 24 H:17 M:7 Age 26 to 51 months	EG: PT sessions and 3 additional body weight supported treadmill training sessions CG: PT sessions consisting of therapeutic activities to promote functional stability and mobility	**Gait:** 10 min gait test **Gross Motor Skills:** GMFM dimensions D and E	**Type:** aerobic **Mode:** Treadmill **Frequency**: three days a week for 6 weeks **Duration:** 15 min a day **Intensity:** Treadmill speed 0.54 to 0.80 m/s, tilt from 0 to 1 degree **Progression:** The speed increased to tolerance in each session, and the maximum reached was the initial speed for the next session **Intervention applied by: a** professional	There were no significant differences in any of the dimensions assessed

GMFM: Gross Motor Function Measure; EG: experimental group; CG: control group.

### Frequency, intensity, and duration of the interventions used in this population

In those studies that applied aerobic therapeutic exercises using the treadmill (mode), the frequency ranged from three days^42^ to five days a week^19,33,35,40,41^.

The duration of each session varied between six^35,38,43^, eight^33,41,43^ and fifteen minutes^42^. The intensity was determined by the treadmill’s speed, which from 0.2 m/s^33,41^, 0.5 m/s^35,37,38^, and 0.54 to 0.80 m/s^42^.

With regard to the person who applied the intervention, this was carried out by professionals in the case of the studies by Lowe, L.^42^ and Angulo-Barroso, R^43^. In Looper, J.^33^, Wu, J.^35^, and Ulrich, D. A.^41^ studies, parents were trained to apply the intervention at home.

Harris SR et al.^40^ assessed an intervention that was different from the aerobic exercise. They applied neuromuscular exercise with a frequency of 3 times a week for 9 weeks, 40 min a day. This intervention was carried out by parents at home after receiving previous training.

### Outcomes assessed in the studies included in the review

Of the outcomes proposed for assessment, no evidence was found for the executive function, balance, and fine motor outcomes in this population. Table 2 includes the features of the studies included in this review.

#### Gait

**Figure 4:**
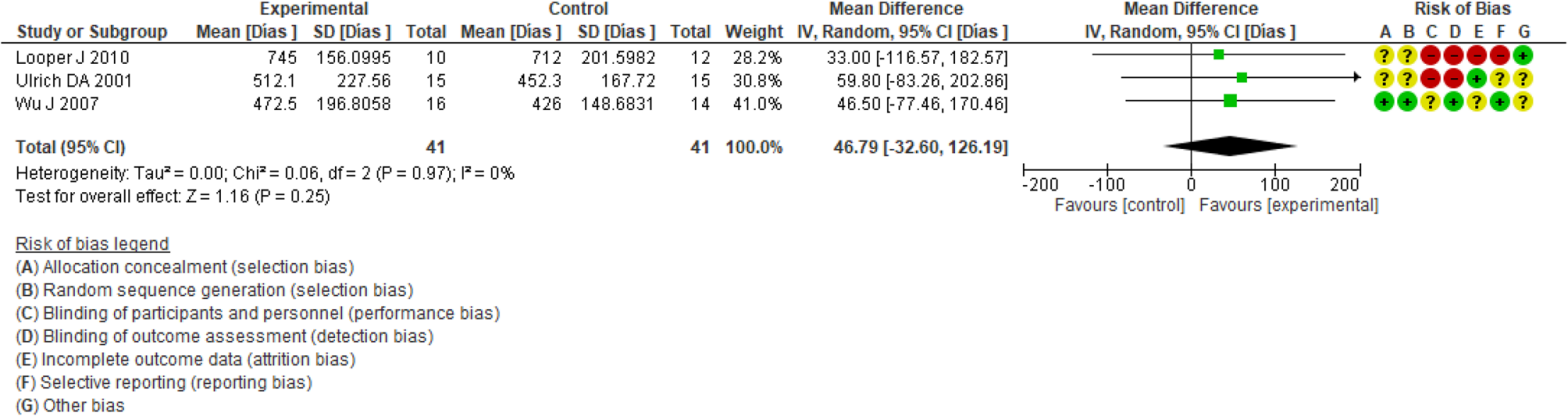
Aerobic exercise (treadmill) versus control (activities of daily living) outcome: independent gait, time(days)-to-event.

Five studies reviewed the effect of therapeutic exercise on the participants’ gait. Angulo-Barroso^19^, Looper, J.^33^, Wu, J.^35^, and Ulrich, D. A.^41^ studies included the average time to achieve independent gait (Fig. 4). Wu, J.^35^ included 30 children with an average age of 10 months. These participants were included in the study when they could remain seated for 30 s. The outcome they assessed was the time to achieve independent gait and kinematic parameters of gait (speed gait), as in the study published by Angulo-Barroso^19^. Finally, Lowe, L.^42^ included 24 participants in his study, with ages that ranged from 26 to 51 months, with the aim of assessing gait performance using the ten-minute gait test however this study not found statistically significant differences between the 4 and 6 weeks of intervention in speed and gait independence. No differences were identified between the treadmill and the physiotherapy plan for the reduction of the time to reach independent walking, mean difference (MD) 46.79, 95% confidence interval (IC) (− 32.60, 126.19), nor for the increase in walking speed MD 0.10 IC (− 0.02, 0.21) m/s.

#### Motor development

Five studies included motor development or motor function as outcome, assessed using scales, such as the Bayley Scales of Infant Development^44^, Gross Motor Function Measure (GMFM)^45^, and Peabody Developmental Motor Scale^46^. Looper, J.^33^ and Lowe, L.^42^ assessed the outcome using the GMFM scale, while Wu, J.^35^ and Ulrich, D. A.^41^ used the Bayley Scales of Infant Development for this purpose. Finally, Harris, S. R.^40^ used the Peabody Developmental Motor Scale.

### Assessment of the certainty of the evidence identified

The certainty of the evidence for the gait and motor development outcomes waslow and low-moderate, respectively (Table 3)^42^.

**Table 3:** Assessing the certainty of the evidence presented for each outcome.

No. of studies	Study design	Risk of bias	Inconsistency	Indirect evidence	Imprecision	Other considerations	Therapeutic exercise	Placebo	Absolute (95% CI)	Certainty
Independent gait: three steps without assistance. Treadmill-type aerobic Therapeutic exercise vs. control (assessed: days-to-event)										
3	Randomized trials	Very serious ^a^	Not serious ^b^	Not serious	Serious ^b^	No effect was observed	41	41	MD **46.79 days** less (32.6 lower than 126.19 higher.)	⨁⨁◯◯ LOW
Motor development (mental dimension) intervention: neuromuscular exercise vs control (regular interventions) (assessed: Gross motor function measure)										
1	Randomized trials	Not serious	–	Serious ^c^	Serious ^b^	No effect was observed	10	10	MD **5.28** less (14.07 less than 3.51 higher.)	⨁⨁◯◯ LOW
Motor development (motor dimension): intervention: neuromuscular exercise vs control (regular interventions) (assessed: Gross motor function measure)										
1	Randomized trials	Not serious	–	Serious ^c^	Serious ^b^	No effect was observed	10	10	MD **5.54 less** (18.01 less than 6.93 higher.)	⨁⨁◯◯ LOW
Gait speed follow-up 4 and 6 weeks intervention: aerobic exercise-weight-supported treadmill training vs regular intervention										
1	Randomized trials	Serious ^d^	–	Not serious	Serious ^b^	No effect was observed	12	12	MD **0.1 higher** (-0.02 lower than 0.21 higher.)	⨁⨁◯◯ LOW
Motor development follow-up 4 and 6 weeks intervention: aerobic exercise-weight-supported treadmill training vs regular intervention (assessed with: GMFM)										
1	Randomized trials	Serious ^d^	–	Not serious	Not serious ^b^	No effect was observed	12	12	MD **2.07 higher** (0.28 higher than 3.86 higher.)	⨁⨁⨁◯ MODERATE

MD: mean difference. ^a^The methods of randomization and the blinding of the evaluators are not clear. There is selective and incomplete reporting of results in one of the studies and doubt in the others; ^b^Confidence intervals are wide; they cross the line of no effect. ^c^Population with intellectual disability including Down Syndrome. ^d^The methods of randomization are not clear.

## Discussion

This is the first systematic review identified in the literature reviewed aimed at assessing the effectiveness of interventions framed within therapeutic exercise (aerobic, resistance, neuromuscular, or neuromotor)^25^ in children aged 0 to 3 years, the stage at which therapeutic interventions are focused on promoting the occurrence of adequate motor patterns^7^. This study found that exercise therapy is effective in improving gait and motor development in children with DS when is compared with activities of daily living. No differences were identified in the mode of application. This study suggests that aerobic exercise therapy has a potentially effective when it is applied using a treadmill with a frequency of 5 days, a duration of 6–8 min, and an intensity of between 0.2 and 0.5 m/s.

Bearing in mind that motor function is a construct that encompasses multiple outcomes and that therapeutic exercise interventions under prescription parameters may favor one outcome over another^47^, according to development stage of children. It intended to identify responses in the literature that could provide better clinical decisions about which type of intervention to use and effective prescription parameters to achieve successful outcomes of interest that will ultimately become the therapeutic goals of clinical interventions.

The evidence identified was scarce in terms of interventions and selected outcomes and their quality. Although they corresponded to randomized clinical experiments, they presented high risk and unclear risk of bias in aspects that jeopardize the internal validity of the study and therefore the certainty when measuring the effect, for example, in the random allocation^33,35,35,40,41^, in the concealment^33,35,35,41^, in the selective data reporting ^33^, and in the follow-up losses, which could lead to selection bias^19,40^. Furthermore, the sample sizes were small, which may explain the width of the confidence intervals and the insignificant differences reported by some studies^40,42^.

The evidence identified corresponds to the same group of authors who, in addition, have carried out a number of thread publications as secondary analyses of the studies carried out, published more than once in different journals (Table 1), which is the reason why these types of publications needed to be independently identified, reported, and not included in the quantitative analyses. New evidence is required, with larger sample sizes and better quality to validate the reported results.

The literature reviewed showed interventions that can be classified into two main types of exercise: aerobic and neuromuscular. Regarding the outcomes outlined in the review, evidence could only be found for the gait and motor development outcomes. For the gait outcome, there is evidence supporting the use of aerobic therapeutic treadmill exercise. This type and mode of exercise was used in five of the six studies identified in this review. After training, parents were in charge of administering the intervention, which consisted of providing stimulation of the gait pattern in children who had not developed the pattern^33,35,38,43^, following previously established parameters. Primary studies showed statistically significant differences in the time-to-the independent gait-event when applying the intervention with a frequency of 5 days, a duration of 6–8 min, and an intensity of between 0.2 and 0.5 m/s.

These findings validate the use of the treadmill as an application mode that can be used in rehabilitation centers for children with DS, as a strategy included in the set of interventions carried out in physiotherapy to promote gait patterns. In the identified evidence, parents applied the intervention at home, which could suggest the use of this intervention as an adjunct to the interventions carried out in rehabilitation centers. However, before recommending its use at home, budget impact and cost-effectiveness analyses would be required to determine whether the benefits achieved would justify the cost of including these interventions^48^.

Only one study applied the intervention with the aim of enhancing the gait patterns of children aged between 26 and 51 months^42^. In this case, no significant differences were found that resulted from the intervention, which may be explained by the frequency and duration parameters, as the frequency was three days a week and 15 min a day. Another explanation for these results could be the small size of the sample, which could result in a type 2 error^49^. Another reason could be the selection bias since there was a difference in the number of girls and boys included and because the population included children with DS and cerebral palsy, among others, and the authors did not carry out a subgroup analysis^50^.

The other outcome reported in literature was motor development. This was the purpose of studies that included aerobic exercise using a treadmill^19,33,35,41,42^ and neuromuscular exercise^40^. Significant differences were reported when using the parameters.

Only one study reported the application of this type of exercise to improve the motor development in children with DS^40^. The authors did not report significant differences in the outcome measured using the Bayley and Peabody Scales. There is evidence of the effectiveness of this type of exercise in improving the balance in older children with DS^22,23^; however, this outcome was not measured in the aforementioned study.

There are innumerable interventions regularly used in physical rehabilitation in institutions treating children with DS that include rehabilitation approaches such as Bobath and Vojta, among others. Hydrotherapy and hippotherapy interventions are also offered in the management of these children. Surprisingly, there is no good-quality evidence to support the use of these modalities^51^. Interventions such as hydrotherapy or aquatic therapy, which has been proven to be effective in improving clinical variables in other populations^52,53^, did not provide evidence that could support their use in the subject population of this review.

Future studies are expected to assess the effects of interventions that are currently used with robust research designs. New evidence is required that increases certainty regarding the measurement of the effects achieved by the studies herein reported. Additionally, it is important to include budget impact and cost-effectiveness analyses for the interventions mentioned herein.

## Limitations of the study

One limitation of the study is the low number of studies that fulfilled the eligibility criteria in terms of outcomes. Therefore, future studies may yield different results for the outcomes posed in this review. The small number of studies was also reported by the authors themselves, which does not allow for a comparative analysis between prescription parameters and even the mode of application of the exercise.

No studies in children aged less than nine months were identified.

## Conclusions

There is low and moderate evidence to support that exercise therapy promotes the occurrence of motor patterns such as gait patterns and improves the motor skills in children with DS aged 0 to 3 years. More common type and mode of exercise reported to improve motor function in these children is aerobic therapeutic treadmill. Motor development could improve if the interventions are made in therapeutic facilities and home. Standardizing the instruments that measure outcomes in motor function and development can help to refine the parameters of exercise prescription and evaluate the effect of intervention.

Future research is required to support the use of effective prescription parameters of the many interventions currently employed in care settings within this population.

## Supplementary Information

Supplementary Information 1.

Supplementary Information 2.

## Abbreviations

DS: Down syndrome
GMFM: Gross motor function measure
GRADE: Grading of recommendations assessment, development and evaluation
PICO: Population, intervention, comparison and outcome
