# Supplementary material for: Therapeutic exercise to improve motor function among children with Down Syndrome aged 0 to 3 years: a systematic literature review and meta‑analysis
Source: Sci Rep. 2022 Jul 29;12:13051. doi: 10.1038/s41598-022-16332-x (PMC9338268; doi:10.1038/s41598-022-16332-x)
Supplement: Supplementary file 2 — Supplementary Information 2. [file 41598_2022_16332_MOESM2_ESM.docx]

| **Data base** | **Algorithms** | **Results** | **Date** |
| --- | --- | --- | --- |
| EMBASE | down syndrome'/exp OR 'down syndrome' OR mongolism OR trisomy AND 21 AND ('infancy'/exp OR 'infancy') OR 'adolescent'/exp OR 'adolescent' OR 'child'/exp OR 'child' AND ('therapeutic exercise'/exp OR 'exercise') OR aerobic AND exercise OR resistance AND training OR physical AND therapy OR physical AND activity OR therapeutic AND exercise OR resistance AND training OR plyometric AND exercise OR muscle AND stretching OR anaerobic OR biciclyng OR aquatic AND exercise executive AND functions OR executive AND control OR executive AND controls OR mental AND processes OR anticipation OR psychological AND cognition OR consciousness OR metacognition OR intention OR decision AND making OR attention OR cognition OR memory OR problem AND solving OR attentions OR cognitions OR cognitive AND function OR cognitive AND functions OR memory OR spatial AND memory AND activities AND of AND daily AND living OR adl OR daily AND living OR living AND activities. | 159 | 09.05.19 |
| Scielo | ((((sindrome de down) AND ( ejercicio físico) OR (entrenamiento ) OR (ejercicio terapéutico) OR (actividad física) OR (deporte)) AND NOT (audición) AND NOT (lenguaje)) AND NOT (comunicativas)) AND NOT (medicamentos) AND NOT (cirugias) AND NOT (farmacos) | 143 | 14.05.19 |
| Epistemó nikos | (title:(down syndrome) OR abstract:(down syndrome)) AND (title:(exercise) OR abstract:(exercise)) OR (title:(aerobic) OR abstract:(aerobic)) OR (title:(resistance) OR abstract:(resistance)) OR (title:(swimming) OR abstract:(swimming)) OR (title:(running) OR abstract:(running)) OR (title:(trendmill) OR abstract:(trendmill)) OR (title:(cycling) OR abstract:(cycling)) OR (title:(training) OR abstract:(training)) OR (title:(sport) OR abstract:(sport)) OR (title:(therapy exercise) OR abstract:(therapy exercise)) OR (title:(physical activity) OR abstract:(physical activity)) | 378 | 09.05.19 |
| Lilacs | (tw:(Síndrome de Down)) AND (tw:(función motora)) AND (tw:(rehabilitación)) | 5 | 09.05.19 |
| Cochrane library | down syndrome and exercise and aerobic | 17 | 14.05.19 |
